# Supplementary material for: Maternal Risk Factors Associated with Negative COVID-19 Outcomes and Their Relation to Socioeconomic Indicators in Brazil
Source: Healthcare (Basel). 2023 Jul 20;11(14):2072. doi: 10.3390/healthcare11142072 (PMC10379003; doi:10.3390/healthcare11142072)
Supplement: Supplementary file 1 [file healthcare-11-02072-s001.zip › healthcare-2455800-supplementary.pdf]

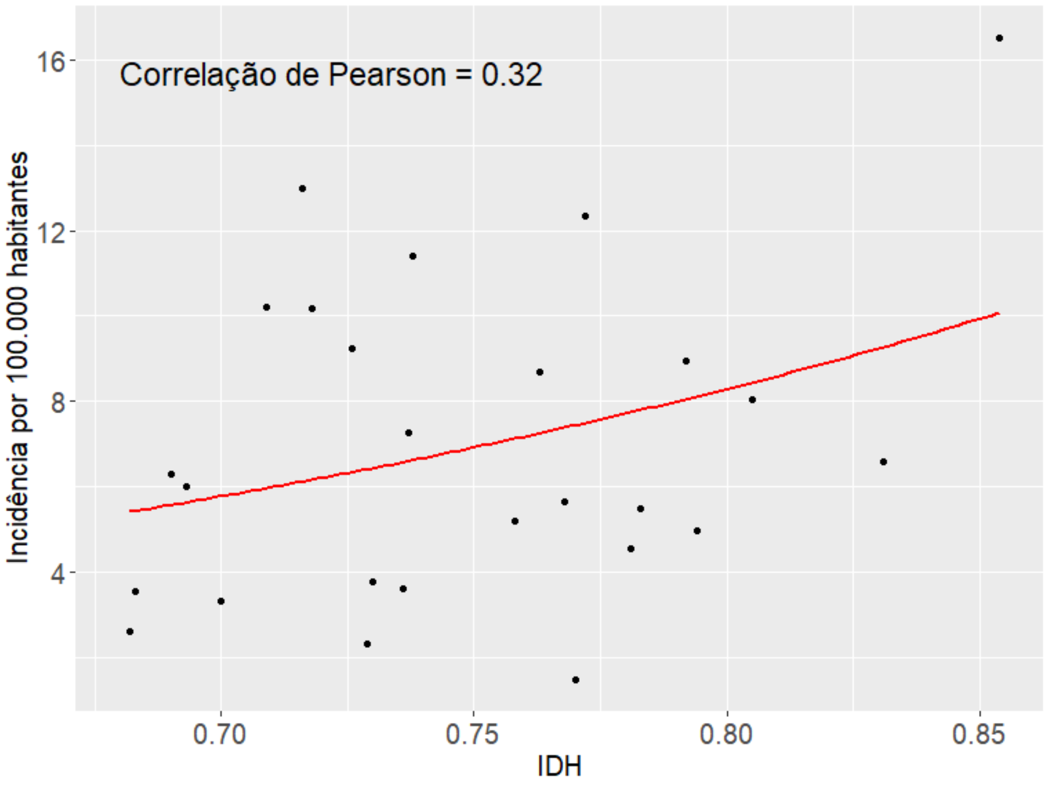

Figure S1: Poisson model adjusted to the points of incidence of Covid-19 cases in pregnant women by the HDI of the state

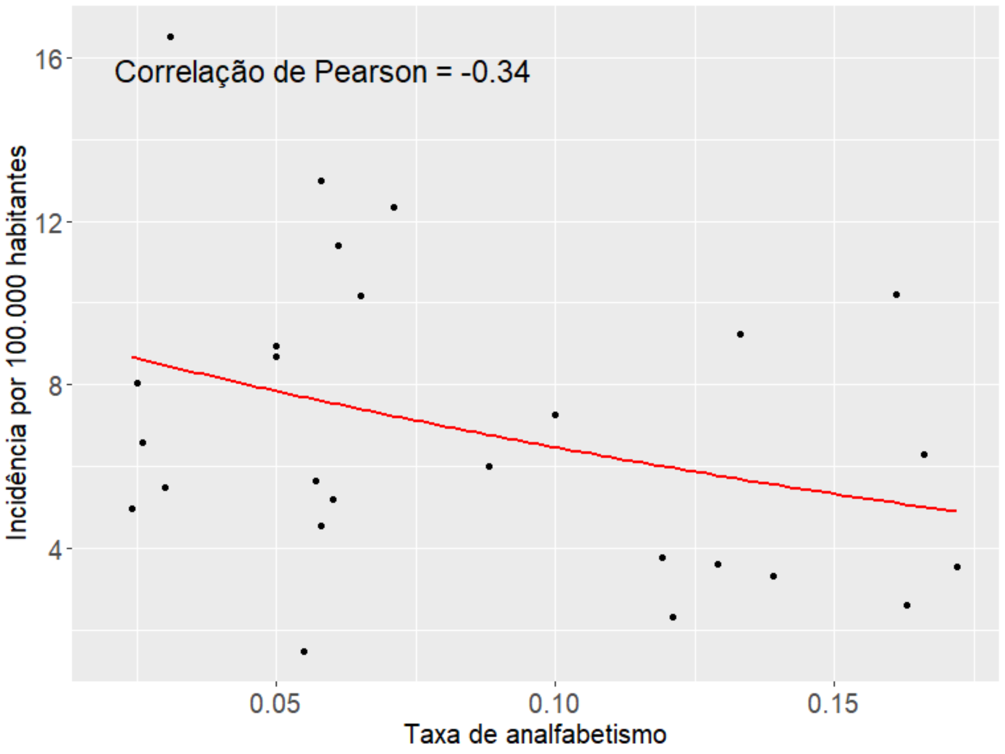

Figure S2: Poisson model adjusted to points of incidence of Covid-19 cases in pregnant women by state illiteracy rate.

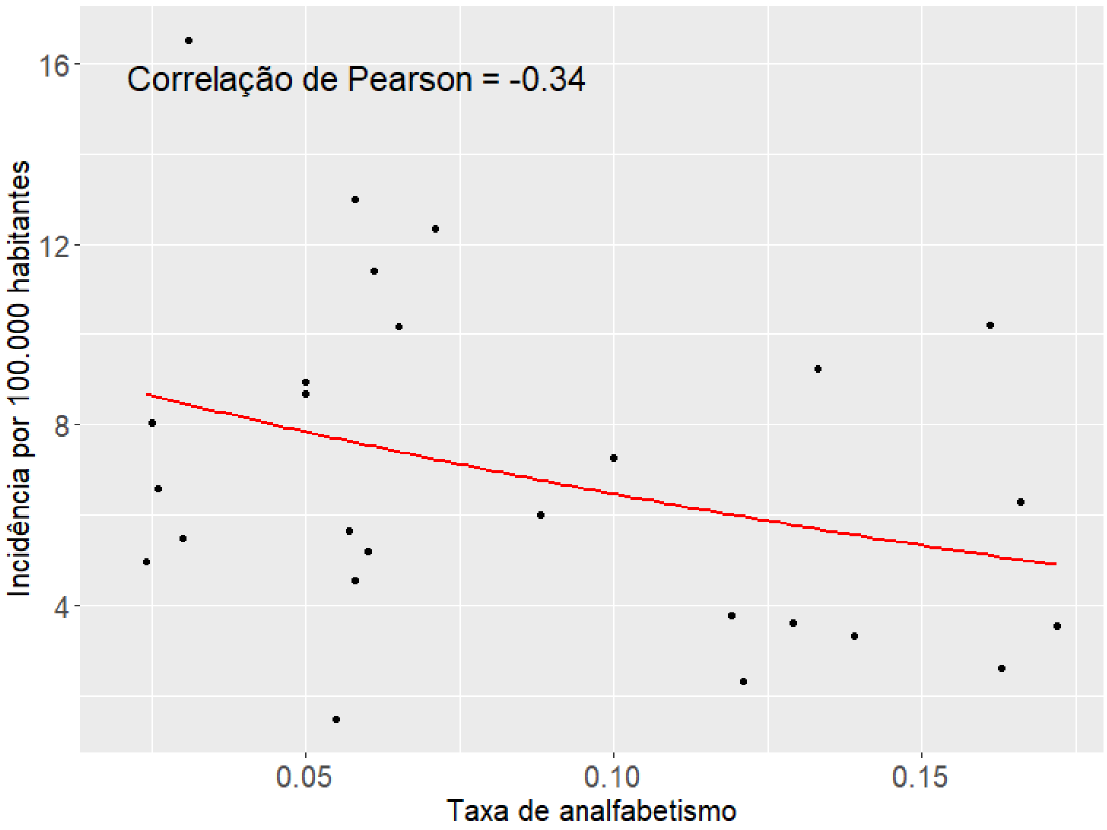

Figure S3: Poisson model adjusted to points of incidence of Covid-19 cases in pregnant women by per capita income in the state.

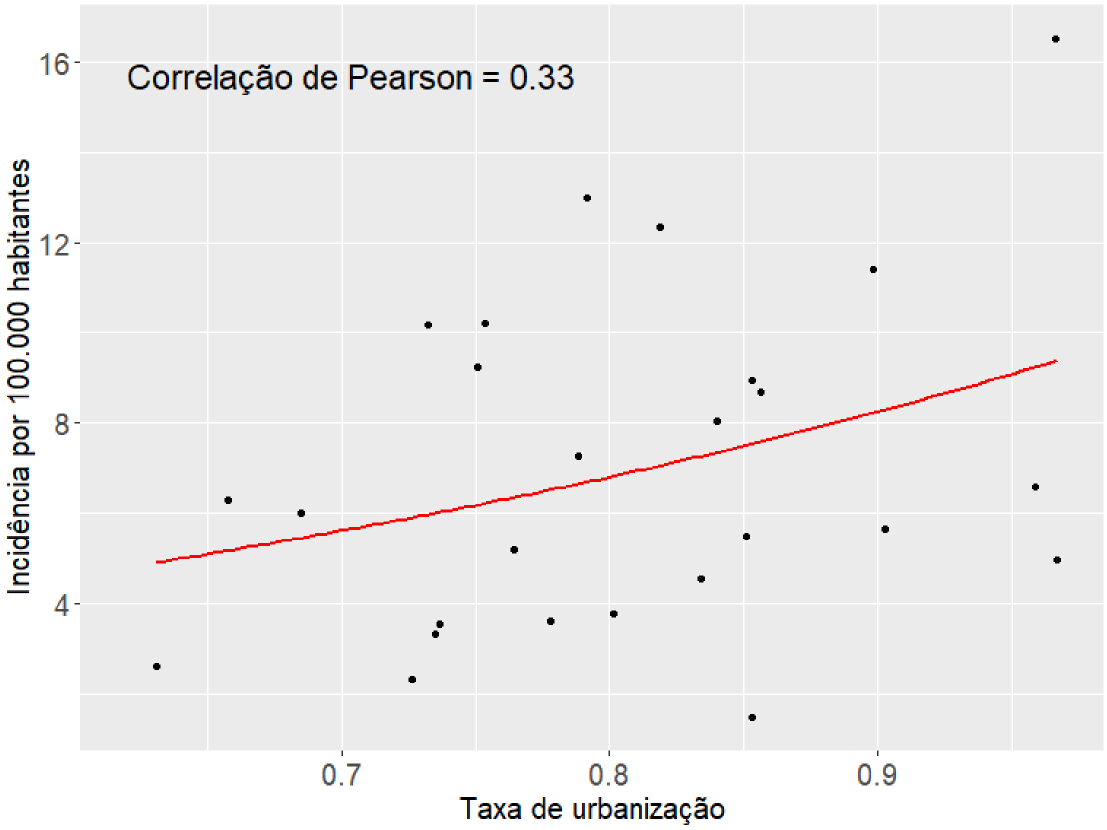

Figure S4: Poisson model adjusted to points of incidence of Covid-19 cases in pregnant women by state urbanization rate.
